# Alterations in Gene Pair Correlations as Potential Diagnostic Markers for Colon Cancer

**DOI:** 10.3390/ijms232012463

**Published:** 2022-10-18

**Authors:** Bonnie Yang Yang, Meena Kishore Sakharkar

**Affiliations:** 1 Department of Anatomy, Physiology and Pharmacology, College of Medicine, University of Saskatchewan, 107 Wiggins Road, Saskatoon, SK S7N 5E5, Canada; 2 Drug Discovery and Development Research Group, College of Pharmacy and Nutrition, University of Saskatchewan, Saskatoon, SK S7N 5E5, Canada

**Keywords:** colon cancer, differentially expressed gene, gene pair correlation, diagnostic marker, signaling pathway

## Abstract

Colorectal cancer (CRC) is a leading cause of death from cancer in Canada. Early detection of CRC remains crucial in managing disease prognosis and improving patient survival. It can also facilitate prevention, screening, and treatment before the disease progresses to a chronic stage. In this study, we developed a strategy for identifying colon cancer biomarkers from both gene expression and gene pair correlation. Using the RNA-Seq dataset TCGA-COAD, a panel of 71 genes, including the 20 most upregulated genes, 20 most downregulated genes and 31 genes involved in the most significantly altered gene pairs, were selected as potential biomarkers for colon cancer. This signature set of genes could be used for early diagnosis. Furthermore, this strategy could be applied to other types of cancer.

## 1. Introduction

Colorectal cancer (CRC), which includes both colon cancer and rectal cancer, is the third most common type of cancer in Canada [1]. According to the Canadian Cancer Society, about 24,300 Canadians were diagnosed with CRC in 2022 (~10% of all new cancer cases) [2]. Globally, it has been estimated that there could be more than 2.2 million new cases and 1.1 million deaths from CRC by the year 2030 [3]. A significant number of CRC cases are sporadic, and their etiology has been linked to lifestyle and environmental factors such as age, ethnicity, sex, diet, morbidity (diabetes or obesity), and tobacco usage [3]. However, 2–8% of CRC cased are due to inherited syndromes [3].

Current treatment options for CRC generally include surgery (endoscopic and local excision) and chemotherapy. Loco-regional surgery, local ablative therapy, targeted therapy, and immunotherapy are also applied for advanced stage and/or metastatic CRC [4]. CRC normally becomes symptomatic in the advanced stage. The five-year survival rate drops from 92% for stage I to 12% for stage IV in colon cancer patients and from 88% for stage I to 13% for stage IV in rectal cancer patients [5]. Thus, early diagnosis is essential for patient survival in CRC. It has also been shown that only about 55.2% of Canadians aged 50–74 have undergone a stool test in the last two years [6] and 62% of Americans aged 50–75 have taken a colonoscopy/sigmoidoscopy in the last 10 years [7]. Although regular colonoscopy has led to a decline in CRC in adults over 50 years old, there is an indication that the CRC rate is increasing among adults less than 50 years old in both Canada [8,9] and the USA [10,11]. A recent study characterized CRC outcomes in young adults and reported that the five-year survival rate was lower in young patients in comparison to middle-aged patients; this has been attributed to advanced tumor stage and distant metastasis at the time of presentation [12].

Up to now, there is no single treatment to treat every patient with equal efficacy [13]. While the development of an effective primary medical intervention is in process, the parallel development of cost-effective, non-invasive early detection techniques (asymptomatic stage) is also under investigation as a preventive strategy in CRC patients. Although extensive genomic and transcriptomic studies have been undertaken to identify differentially expressed genes (DEG) as potential cancer biomarkers [14,15], they have not been significantly impactful. This is because CRC is highly heterogeneous and complex and is dynamically governed by a combination of vast genes and environmental factors [16]. To better characterize CRC, gene expression has to be analyzed as a system rather than an individual event. Herein, we propose that alterations in the correlations for expression of gene pairs could serve as a supplement to the current clinical biomarkers for early disease diagnosis. This novel approach integrates multiple factors to shortlist pharmacologically relevant targets using a set of indirect and network-dependent features that can be modulated in a differential fashion to bring out the required phenotypic effect and help in disease management.

## 2. Results

### 2.1. Differentially Expressed Genes (DEGs)

Gene expression data were extracted from The Cancer Genome Atlas (TCGA) dataset TCGA-COAD. The dataset contains RNA-Seq expression data and the corresponding clinical data for 471 colon cancer patients and 41 healthy controls. Using |log_2_FC| ≥ 2.50 (FC, fold change) as the cutoff, we identified 606 upregulated DEGs and 447 downregulated DEGs (Supplementary Table S1). The top 20 upregulated and downregulated DEGs are summarized in Table 1.

### 2.2. Pathway Analysis

The upregulated and downregulated DEGs (|log_2_FC| ≥ 2.50) were subjected to pathway analysis using InnateDB [17], which can categorize proteins into different biological pathways. The top 10 significant biological pathways for the KEGG Pathways Database were neuroactive ligand–receptor interaction, systemic lupus erythematosus, cytokine–cytokine receptor interaction, pathways in cancer, bile secretion, Wnt signaling pathway, cell adhesion molecules, metabolism of xenobiotics by cytochrome P450, drug metabolism, and tight junction (Table 2). The uploaded gene count, (i.e., number of DEGs) and the total number of genes in the top 10 biological pathways are provided in Table 2.

### 2.3. Alternation in Gene Pair Correlations

**Figure 1:**
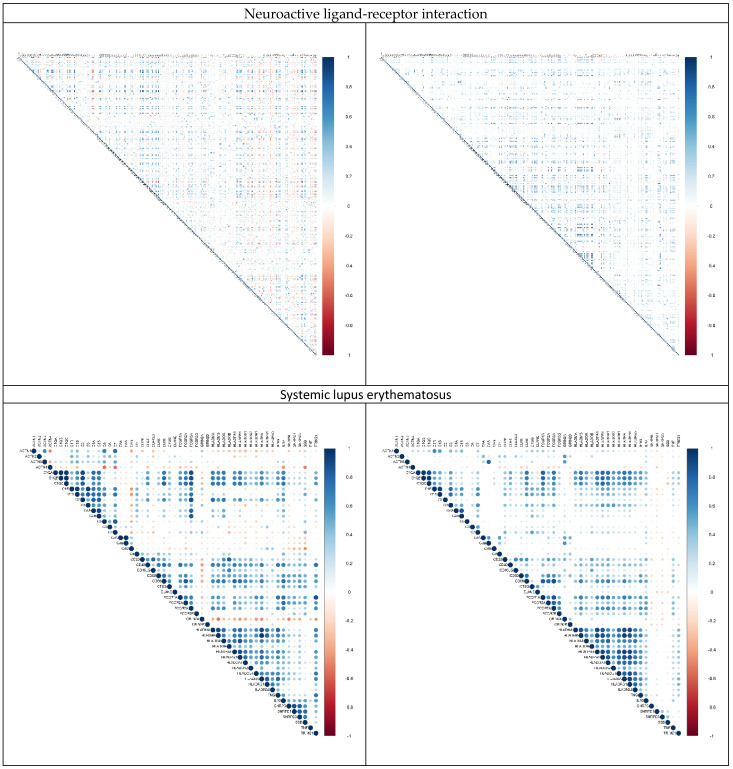

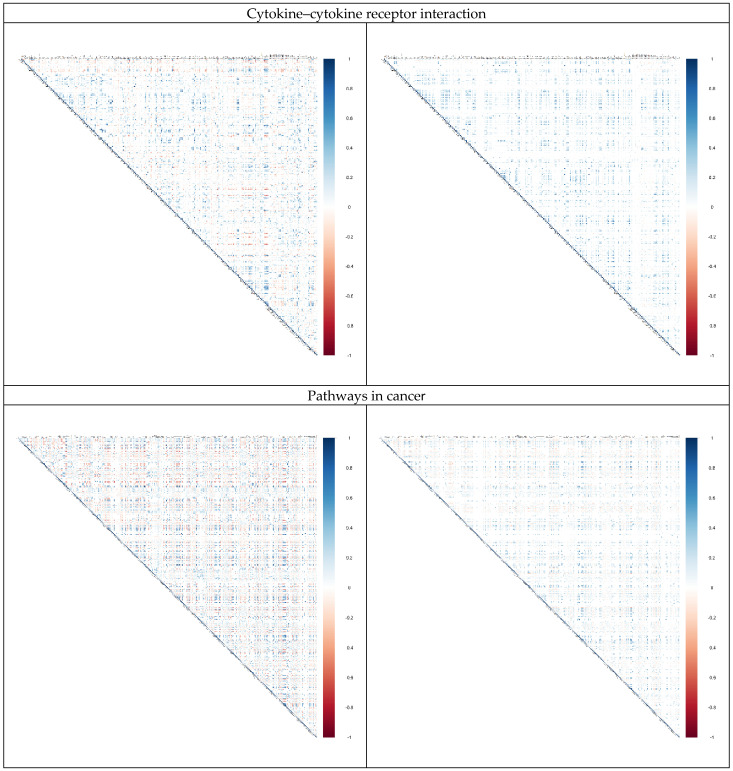

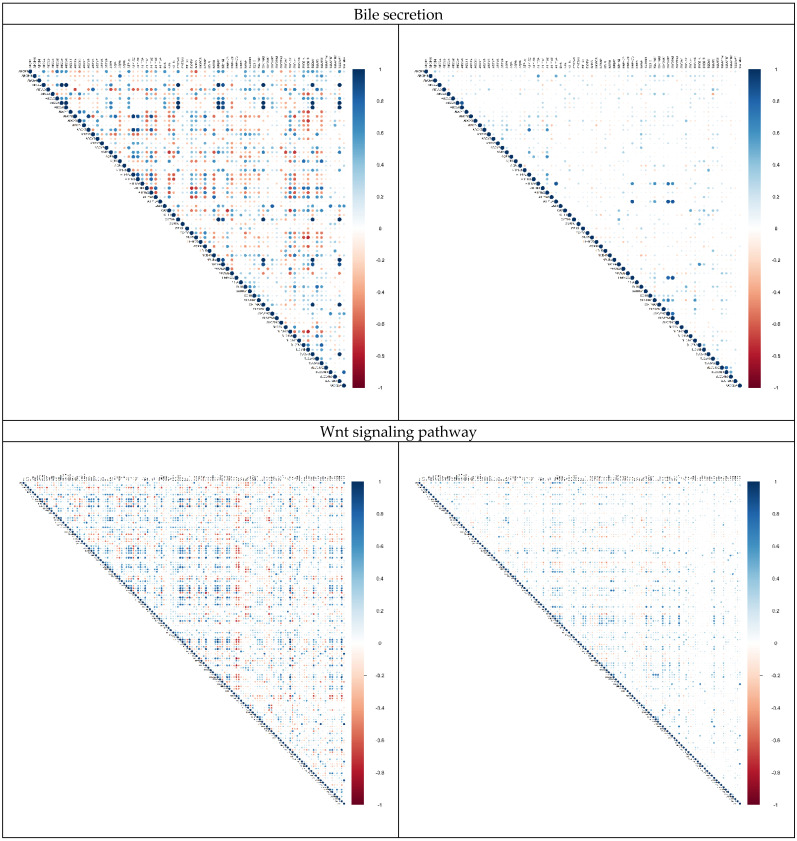

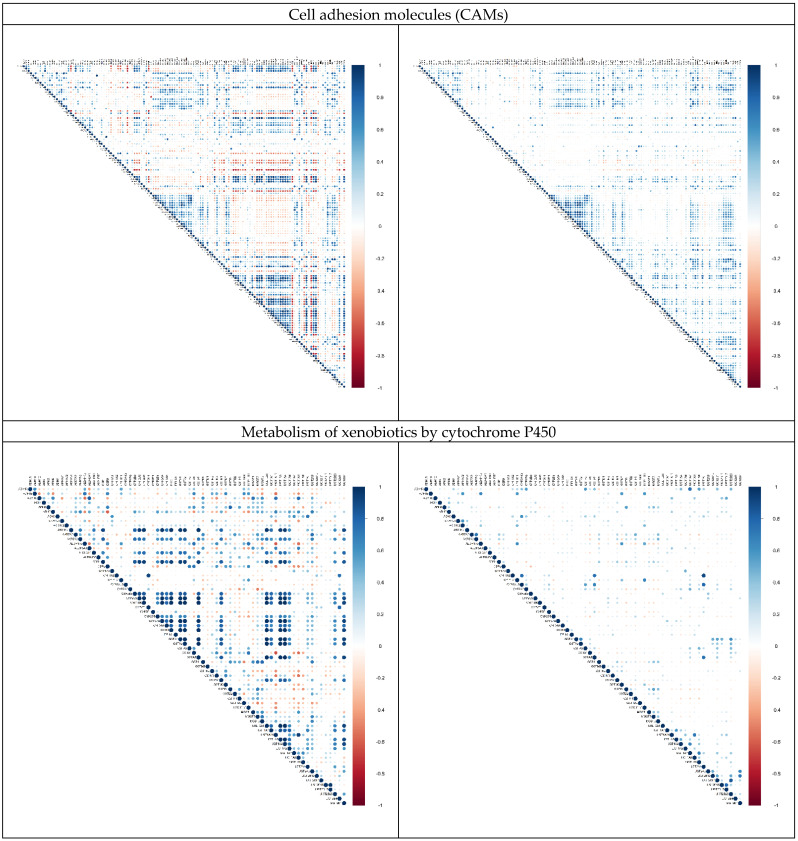

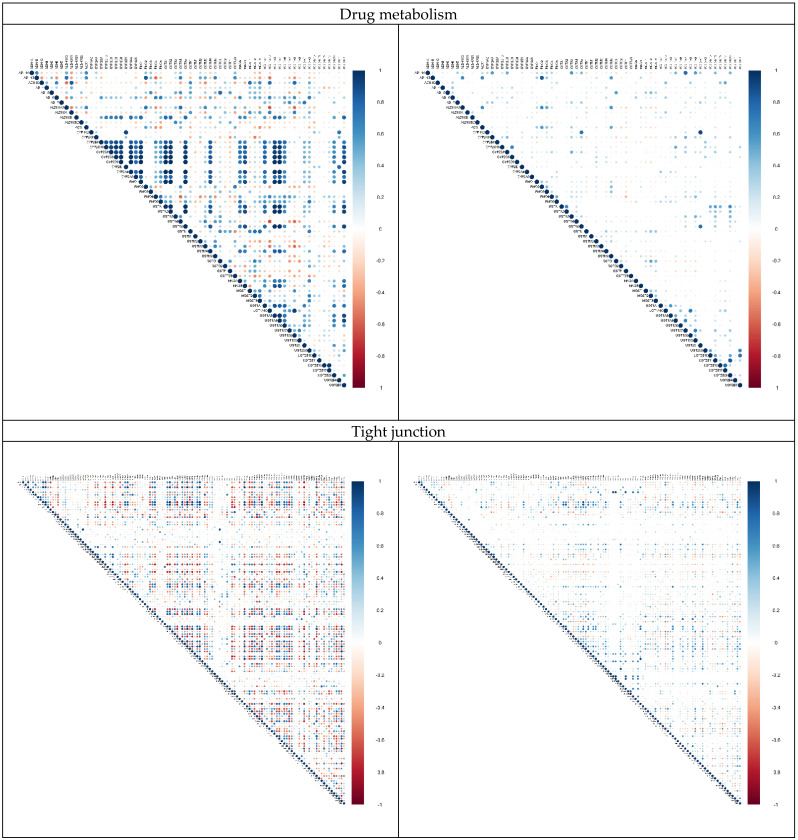
Comparison of the gene pair correlations for genes in the top 10 significant biological pathways between healthy controls (**left**) and colon cancer patients (**right**). The 10 biological pathways are neuroactive ligand–receptor interaction, systemic lupus erythematosus, cytokine–cytokine receptor interaction, pathways in cancer, bile secretion, Wnt signaling pathway, cell adhesion molecules, metabolism of xenobiotics by cytochrome P450, drug metabolism, and tight junction. Positive and negative correlations were shown in blue and red, respectively.

Carcinogenesis is a complicated and complex process, which requires the coordination of multiple genes. To understand the modulation of the top 10 significant biological pathways and the change in expression in relation to each other in colon cancer, we calculated and compared the pairwise correlations for all genes in each biological pathway between healthy controls and colon cancer patients (Figure 1). It is clear that the genes in most of the biological pathways tend to lose their correlations/coordination upon carcinogenesis, implying that the decoupling of gene regulations might be essential for colon cancer development. This observation is consistent with our previous studies [18,19,20].

## 3. Discussion

### 3.1. Top 20 Upregulated DEGs

As shown above, the top 20 upregulated DEGs had their fold changes more than 360 folds. Further analysis showed that four upregulated DEGs belong to the Melanoma-Associated Antigen-A (MAGE-A) cluster. The MAGE-A gene cluster is located at Xq28 in the human genome and consists of 12 genes. The genes show silent/low expression in all normal tissues except in the male germ cell and the placenta, as the MAGE-A family is involved in spermatogenesis and embryonic development. Recently, the role of Melanoma-Associated Antigen-A (MAGE-A) gene expression in various human cancers was reviewed and it was reported that genes in this family are potential therapeutic targets in cancer immunotherapies [21]. Although the exact function of these genes is not clearly understood, some members (MAGE-A2, A3/6, and A9) have been shown to be tumorigenic and involved in the dysregulation of the tumor suppressor p53 [22,23,24,25,26].

Overexpression of *IGF-1* has been linked to an increased risk of colon cancer, and IGF-1 has been shown to stimulate the growth of the colon cancer cell lines HT-29 and SW-480 [27,28]. Three kallikreins, KLK6, KLK7, and KLK8, were found to be overexpressed in colon cancer. The kallikrein gene family, which is located on chromosome 19q13.4, encodes serine proteases. Proteases including kallikreins have been associated with the progression of colon cancer and reported to help in cancer progression by extracellular matrices and the invasion of surrounding tissues by the transformed cells [29,30]. KLK6, KLK7, KLK8, and KLK10 were recently reported as potential diagnostic biomarkers for colon adenocarcinoma [31]. PAEP (Glycodelin) is an immunosuppressive glycoprotein and a high level of glycodelin is observed in the serum of colon cancer patients [32]. It was reported as a biomarker for colon cancer as the serum glycodelin level is increased in patients with metastatic CRC [33]. *NOTUM* encodes palmitoleoyl-protein carboxylesterase, which is involved in the proliferation and migration of colorectal cancer and proposed as a diagnostic and therapeutic candidate [34].

SPRR1A, SPRR2E, and SPRR2D are three members of the small proline-rich proteins (involved in the keratinization pathway). SPRRs are critical components of the cornified cell envelope (CE). The CE is involved in the formation of an envelope beneath the plasma membrane that serves as a barrier to extracellular and environmental factors. Dysregulation results in the compromise of the barrier [35]. PRR9, a paralog of SPRR2G, is also observed to be overexpressed. SPRR1A was recently proposed as a prognostic marker of colon cancer as its expression was higher in tumors compared to the adjacent noncancerous tissues [36]. High expression of SPRR1A was reported to be associated with poor survival in colon cancer patients [37]. SPRR1A and SPRR2D are also interacting partners of KLK6 [38]. PPBP (also known as CXCL7) is a chemokine involved in the immune response during vascular injury [38,39]. It is proposed as a potential biomarker for cancers [40,41,42]. Overexpression of PPBP affects the PI3K/AKT/mTOR signaling pathway and is associated with poor prognosis for colon cancer [43,44,45].

*DKK4* is a member of the Dickkopf family and modulates the Wnt signaling pathway. It was reported to enhance the migration and invasive characteristics of colon cancer cells [46]. Overexpression of *DKK4* in colorectal cancer was also previously reported [47]. Zinc finger of the cerebellum 5 (ZIC5) is a transcriptional repressor. It plays an important role in colorectal cancer via regulating cell cycle progression and the modulation of CDC25/CDK/cyclin signaling [48]. Cystatin SN (cystatin 1, CST1) inhibits cysteine proteases and promotes cell proliferation and metastasis. It was reported as a biomarker in colorectal cancers [49]. *COL10A* is a member of the collagen family and has been reported to be overexpressed in colorectal cancer. It has also been proposed as a potential biomarker [50]. *PRSS56* encodes a serine protease, which has a significant correlation with clinical stage in bile duct cancer [51]. FEZF1 is a transcriptional repressor of the zinc finger double domain protein family. No relationship with *FEZF1* in CRC could be identified in the literature (as of 14 September 2022).

### 3.2. Top 20 Downregulated DEGs

As for the downregulated SDEGs, all of them had fold changes more than 45 folds. Four lipoproteins, APOA4, AOC3, APOA1, and APOB, were downregulated. Lipoproteins are formed by the attachment of apolipoproteins (APOs) to lipids, and hence apolipoproteins function as lipid carriers. APOs are primarily synthesized in the liver and the intestine. APOA4 and APOA1 are involved in the transportation of chylomicrons (low-density lipoproteins, LDLs) and high-density lipoproteins (HDLs) [52], while APOB transports LDLs and APOC3 transports HDLs. APOA4 possesses anti-inflammatory activity in allergies [53]. However, no report was found on its association with colon cancer or CRC. Low ApoA-I levels have been proposed to be associated with increased mortality risk in colorectal cancer patients. APOA1 was reported to be associated with anti-inflammatory, antiangiogenic [54], immunoregulatory [55,56], and antithrombotic [57,58] activities. Although no association was found between colon cancer and APOB and APOC3 expressions, a recent report evaluated the role of APOB in bile duct cancer and demonstrated that APOB influences the infiltration degree of immune cells [59]. Furthermore, APOC3 is involved in the breakdown of triglycerides, and hence its plasma level directly correlates with the plasma triglyceride level [60].

*OTOP2* and *OTOP3* encode for otopetrin 2 and otopetrin 3, respectively. *OTOP2* was downregulated in CRC and its expression is negatively correlated with the malignancy grade and patient survival [61]. Although the exact mechanism of action is not known, it could be regulated by wild type p53 [62]. OTOP3, which is a paralog of OTOP2, functions as a proton-selective channel and is involved in the maintenance of intracellular pH. OTOP3 has been reported as an interesting candidate for future research in colon cancer [63].

SLC10A2 (ASBT/Apical sodium-dependent bile acid transporter) and SLC30A10 (Solute Carrier Family 30 Member 10) are also downregulated in colon cancer. ASBT is involved in the reclamation of bile acids at the terminal ileum enterocyte brush border membrane [64]. ASBT-deficient mice are reported to show a 2-fold increase in the number of colon adenocarcinomas [65]. SLC30A10 has been observed to be downregulated in colorectal cancer tissues and cell lines. Low expression of SLC30A10 promotes cell proliferation and migration of colorectal cancer cells [66]. SLC30A10, which is a Mn transporter, is also involved in Zn transport in endosomes. Mn is essential for the function of several enzymes, such as those involved in the metabolism of neurotransmitters. The loss-of-function mutations of *SLC30A10* are associated with adult-onset Parkinsonism. Expression of *SLC30A10* and cellular Mn efflux are regulated by the composition of bile acids [67]. 

*MS4A10* encodes for a component of a multimeric receptor complex, which is involved in signal transduction. However, its exact function is unknown. AQP8 is an aquaporin involved in water transport across the biological membranes. It is downregulated in CRC. AQP8 inhibits PI3K/AKT signaling [68]. CA1 encodes for carbonic anhydrase 1 and is reported to be downregulated in CRC patients [69]. *CA1* is involved in electroneutral sodium chloride reabsorption and short-chain fatty acid uptake [69]. *INSL5* belongs to the insulin superfamily and is downregulated in colon cancer. It encodes insulin-like peptide 5. Recently, *INSL5* was reported as a potential therapeutic target for CRC [70]. It also plays a role in gut contractility in murine models [71]. It is important to note that colon cancer patients usually show changes in bowel movements including constipation. TMIGD1, which is a putative tumor suppressor, can induce G2-M cell cycle checkpoint arrest in colon cancer cells and is correlated with poor overall survival [72]. *GUCA2B* encodes uroguanylin, which is an endogenous hormone functioning as a paracrine endogenous ligand to regulate proliferation, metabolism, and barrier function in the intestine by binding and activating guanylate cyclase C (encoded by *GUCY2C*) [73].

*G6PC* is a glucose-6-phosphatase catalytic subunit component gene. Glucose-6-phosphatase is involved in gluconeogenesis and glycogenolysis. Thus, it plays a key role in glucose homeostasis. Alterations in glucose metabolism have been noticed in many types of cancer, including colon cancer [74]. *CPO* encodes carboxypeptidase O, which is involved in the small intestine phase of protein digestion. It is a membrane-anchored brush-border enzyme that enables the C-t proteolysis of the great majority of amino acids present in dietary proteins [75]. *CPO* was observed to be downregulated in our current analysis and no report on its downregulation in either colon cancer or CRC was found in the literature. *KRTAP13-2* encodes Keratin Associated Protein 13-2, which was also downregulated in rectal cancer [76]. Peptide YY (encoded by *PYY*) is a gut hormone. Decreased expression of peptide YY has been reported to be relevant to the development and progression of colon adenocarcinoma [77]. *BEST4* is significantly expressed in the colon and belongs to the bestrophin gene family of anion channels. However, it is dramatically downregulated in CRC [78]. Claudin 8 (encoded by *CLDN8*) is part of the tight junction in cell membranes. In contrast to our observation, CLDN8 was previously reported to be overexpressed in CRC patients and promoted cell proliferation, migration, and invasion of colorectal cancer cells [79].

We herein provide a brief overview on the functions of the top 40 modulated DEGs in colon cancer patients. Several genes have been identified as targets to develop therapeutic agents against colon cancer. However, early detection remains crucial for cancer patient survival, despite recent progress in drug development. Although several biomarkers and targets have been proposed for colon cancer (some of them were discussed above), there is lack of a technique that can address the issue of heterogeneity and differential expression of biomarkers in colon cancer in a unified and effective manner among diverse populations. Here, we investigated the alterations in gene pair correlations of the top 10 regulated biological pathways in colon cancer and identified a set of genes that could be applied for the early diagnosis of colon cancer.

### 3.3. Alternation in Gene Pair Correlations of the DEGs

In order to identify potential genes that could be used for the early detection of colon cancer, we calculated the changes of gene pair correlation coefficients (CCs) between colon cancer patients and health controls and identified a set of gene pairs with |log_2_FC| ≥ 2.50 and |ΔCC| (i.e., CC_cancer_ − CC_control_) ≥ 0.70.

***Neuroactive ligand–receptor interaction***: The 29 DEGs involved in this pathway are *ADCYAP1R1*, *AGTR1*, *CHRM2*, *CNR1*, *CTSG*, *DRD2*, *F2*, *GABRD*, *GABRE*, *GABRG2*, *GABRP*, *GLP2R*, *GRIK3*, *GRIN2B*, *GRIN2D*, *GRPR*, *HTR1D*, *HTR4*, *KISS1R*, *NMUR2*, *NPFFR1*, *NPY2R*, *OXTR*, *P2RX2*, *P2RY4*, *PRSS1*, *PRSS3P2*, *SSTR5*, and *TACR2*. However, none of these genes were among the top 20 up- or downregulated genes. Using |ΔCC| ≥ 0.70, we identified three gene pairs, *CNR1*-*CHRM2* (−0.71), *GRPR*-*AGTR1* (−0.87), and *GRPR*-*TACR2* (−0.78). The log_2_FC for CNR1, CHRM2, GRPR, AGTR1, and TACR2 were −3.20, −3.00, 3.13, −3.30, and −3.40, respectively.

***Systemic lupus erythematosus***: The 28 DEGs involved in this pathway are *C7*, *CTSG*, *ELANE*, *GRIN2B*, *HIST1H2AD*, *HIST1H2AH*, *HIST1H2AJ*, *HIST1H2AM*, *HIST1H2BE*, *HIST1H2BF*, *HIST1H2BI*, *HIST1H2BL*, *HIST1H2BO*, *HIST1H3D*, *HIST1H3F*, *HIST1H3I*, *HIST1H3J*, *HIST1H4A*, *HIST1H4B*, *HIST1H4C*, *HIST1H4D*, *HIST1H4E*, *HIST1H4L*, *HIST2H2AB*, *HIST2H2AC*, *HIST2H2BF*, *HIST2H3D*, and *HIST3H2BB*. None of the 28 DEGs belong to the top 20 up- or downregulated genes. Twenty-five of these genes are in the histone cluster. This is consistent with epigenetic alterations, such as DNA methylation and histone acetylation, contributing to changes in gene expression in systemic lupus erythematosus [80]. However, no gene pair shows a change in correlation of |ΔCC| ≥ 0.70.

***Cytokine–cytokine receptor interaction***: The 27 DEGs involved in this pathway are *AMH*, *BMP7*, *CCL26*, *CNTFR*, *CSF2*, *CXCL11*, *CXCL1*, *CXCL2*, *CXCL3*, *CXCL5*, *CXCL6*, *EDAR*, *FIGF*, *IFNK*, *IL11*, *IL1A*, *IL23A*, *IL6R*, *IL8*, *INHBA*, *LIFR*, *OSM*, *PPBP*, *TNFRSF12A*, *TNFRSF13B*, *TNFRSF17*, and *TNFSF9*. None of the 27 DEGs belong to the top 20 up- or downregulated genes. Only one gene pair, *CXCL5*-*CXCL11* (−0.78), was identified to have |ΔCC| ≥ 0.70. The log_2_FC for *CXCL5* and *CXCL11* were 5.50 and 2.79, respectively.

***Pathways in cancer***: The 19 DEGs involved in this pathway are *AXIN2*, *BIRC7*, *CDKN2A*, *CDKN2B*, *CTNNA3*, *FGF19*, *FGF20*, *FGF3*, *FIGF*, *FZD10*, *IL8*, *MMP1*, *PRKCG*, *RXRG*, *WNT11*, *WNT2*, *WNT3*, *WNT7B*, and *ZBTB16*. None of the 19 genes belong to the top 20 up- or downregulated genes. Two gene pairs, *WNT3*-*FGF19* (−0.82) and *PRKCG*-*CDKN2B* (0.76), met the |ΔCC| ≥ 0.70 cutoff requirement. The log_2_FC for *WNT3*, *FGF19*, *PRKCG,* and *CDKN2B* were 2.64, 3.91, 3.80, and −3.04, respectively.

***Bile secretion pathway***: The 18 DEGs involved in this pathway are *BABCB11*, *ABCG2*, *ADCY5*, *AQP8*, *AQP9*, *ATP1A2*, *BAAT*, *CA2*, *CYP3A4*, *NR1H4*, *SLC10A2*, *SLC4A4*, *SLC51A*, *SLC51B*, *SLC9A3*, *SLCO1B3*, *SULT2A1*, and *UGT2B4*. Four gene pairs, *SLC10A2*-*CYP3A4* (0.77), *SULT2A1*-*CYP3A4* (−0.96), *SULT2A1*-*NR1H4* (−0.86), and *SULT2A1*-*SLC10A2* (−0.98), had |ΔCC| ≥ 0.70. The log_2_FC for *SLC10A2*, *CYP3A4*, *SULT2A1,* and *NR1H4* were −7.16, −4.74, −2.52, and −3.59, respectively. Only *SLC10A2* is in the top 20-downregulated gene list.

***Wnt signaling pathway***: The 17 DEGs involved in this pathway are *AXIN2*, *DKK1*, *DKK4*, *FOSL1*, *FZD10*, *MMP7*, *NKD1*, *NKD2*, *PRKCG*, *SFRP1*, *SFRP4*, *SFRP5*, *WIF1*, *WNT11*, *WNT2*, *WNT3*, and *WNT7B*. The gene pairs *AXIN2*-*NKD1* (0.84) and *WNT7B*-*FOSL1* (0.86) satisfied the |ΔCC| ≥ 0.70 cutoff requirement. The log_2_FC for *AXIN2*, *NKD1*, *WNT7B*, and *FOSL1* were 2.62, 4.41, 6.39, and 2.76, respectively. None of them belong to the top 20 up- or downregulated genes.

***Cell adhesion molecules***: The 17 DEGs involved in this pathway are *CADM3*, *CDH3*, *CLDN10*, *CLDN14*, *CLDN16*, *CLDN1*, *CLDN18*, *CLDN23*, *CLDN2*, *CLDN6*, *CLDN8*, *CLDN9*, *CNTN2*, *NCAM1*, *NEGR1*, *NLGN1*, and *NRXN1*. None of these genes are present in the list of top 20 up- or downregulated genes. Two gene pairs, *CDH3*-*CLDN18* (−0.73) and *CLDN1*-*CLDN14* (−0.70), show a change in correlation of |ΔCC| ≥ 0.70. The log_2_FC for *CDH3*, *CLDN18*, *CLDN1*, and *CLDN14* were 6.12, 7.57, 5.04, and 3.32, respectively.

***Metabolism of xenobiotics by cytochrome P450***: The 16 DEGs involved in this pathway are *ADH1B*, *ADH1C*, *AKR1C4*, *ALDH3B2*, *CYP3A4*, *GSTA1*, *GSTA2*, *GSTM5*, *SULT2A1*, *UGT1A10*, *UGT1A1*, *UGT1A8*, *UGT2A3*, *UGT2B15*, *UGT2B17*, and *UGT2B4*. None of these genes are in the list of top 20 up- or downregulated genes. Using |ΔCC| ≥ 0.70 as the cutoff, we identified 11 gene pairs, *AKR1C4*-*CYP3A4* (−0.97), *AKR1C4*-*GSTA1* (−0.95), *AKR1C4*-*GSTA2* (−0.93), *AKR1C4*-*SULT2A1* (−0.87), *AKR1C4*-*UGT1A1* (−0.72), *CYP3A4*-*GSTA2* (−0.99), *CYP3A4*-*SULT2A1* (−0.96), *GSTA1*-*UGT1A1* (−0.80), *GSTA2*- *UGT1A1* (−0.80), *GSTA2*-*SULT2A1* (−0.99), and *SULT2A1*-*UGT1A1* (−0.78). The 11 gene pairs involve six genes, *AKR1C4*, *CYP3A4*, *GSTA1*, *GSTA2*, *SULT2A1*, and *UGT1A1*, with their respective log_2_FC of 5.83, −4.74, −3.90, −4.79, −2.52, and −2.73. None of them are in the list of top 20 up- or downregulated genes.

***Drug metabolism***: The 14 DEGs involved in this pathway are *ADH1B*, *ADH1C*, *ALDH3B2*, *CYP3A4*, *GSTA1*, *GSTA2*, *GSTM5*, *UGT1A10*, *UGT1A1*, *UGT1A8*, *UGT2A3*, *UGT2B15*, *UGT2B17*, and *UGT2B4*. None of these genes are in our list of top 20 up- or downregulated genes. Two gene pairs, *GSTA1*-*UGT1A1* (−0.80) and *GSTA2*-*UGT1A1* (−0.80), met the |ΔCC| ≥ 0.70 cutoff. The log_2_FC for *GSTA1*, *UGT1A1*, and *GSTA2* were −3.90, −2.73, and −4.79, respectively.

***Tight junction***: The 14 DEGs involved in this pathway are *CLDN10*, *CLDN14*, *CLDN16*, *CLDN1*, *CLDN18*, *CLDN23*, *CLDN2*, *CLDN6*, *CLDN8*, *CLDN9*, *CTNNA3*, *MYH11*, *MYL9*, and *PRKCG*. Only one gene pair, *CLDN1*-*CLDN14* (−0.70), met the |ΔCC| ≥ 0.70 cutoff. The log_2_FC for *CLDN1* and *CLDN14* were 5.04 and 3.32, respectively. Neither of them are in the list of top 20 up- or downregulated genes.

### 3.4. Design an Assay for Potential Early Diagnosis of Colon Cancer

The human body is a complex system. Any physiological process involves the delicate regulation of a system that contains many genes. These genes coordinate one another to fulfill the normal biological and physiological functions. Thus, any pathophysiological process, such as carcinogenesis and cancer development, would involve the disruption of current regulations and/or the re-establishment of new regulations. Our previous studies have shown that genes tend to lose their coordination in different types of cancer. Thus, we undertook the current study in an attempt to develop an assay that could be used for the early diagnosis of colon cancer. From the above analysis, we identified 25 gene pairs, which involve 32 genes, that met the significance criteria of |log_2_FC| ≥ 2.50 and |ΔCC| ≥ 0.70. Although only one gene, *SLC10A2*, is in the list of the top 20 downregulated genes, the other genes play important roles in regulating vast biological, physiological, and/or pathophysiological processes. For example, gene *AGTR1* encodes the angiotensin II receptor type 1. High expression of *AGTR1* will stimulate the expression of vascular endothelial growth factor (VEGF), the key growth factor controlling angiogenesis, and is associated with a poor prognosis for colorectal cancer [81,82,83]. Herein, we propose an assay array, which includes the top 20 upregulated genes, top 20 downregulated genes, and the 31 genes involved in the significantly altered gene pairs, for potential early diagnosis of colon cancer. This array takes both gene expression and gene coordination into consideration. It would be more advantageous over the traditional biomarkers that only involve gene or protein expression. Further studies are warranted to validate and optimize this array using both tissue and fecal samples from colon cancer patients.

## 4. Materials and Methods

### 4.1. Data Acquisition

The colon cancer dataset TCGA-COAD was downloaded from The Cancer Genome Atlas (TCGA) via the Genomic Data Commons (GDC) data portal. It contains the RNA-Seq and clinical information for 471 colon adenocarcinoma patients and 41 healthy controls. For every patient or control, we analyzed the expression of 60,483 RNA transcripts in terms of FPKM values.

### 4.2. Identification and Visualization of Differentially Expressed Genes

Differentially expressed genes (DEGs) were identified using the DEGseq package from R based on a previously published protocol developed in our laboratory [18,19,20]. The output was expressed in normalized Log_2_FC (FC, fold change). Information on the change in expression for the genes in colon cancer patients compared to healthy controls was extracted using *p* < 0.001 and |log_2_FC| ≥ 2.50 as the significance criteria.

### 4.3. Biological Pathway Analysis

A Pathway Overrepresentation Analysis was performed using InnateDB [17], a publicly available resource that predicts biological pathways based on experiment fold change data sets, based on levels of differential gene expression. Pathways were assigned a probability value (P) based on the number of proteins present in a particular pathway and the degree to which they were differentially expressed or modified, relative to a control condition. The top 10 biological pathways that are significantly modulated in colon cancer were identified to be neuroactive ligand–receptor interaction, systemic lupus erythematosus, cytokine–cytokine receptor interaction, pathways in cancer, bile secretion, Wnt signaling pathway, cell adhesion molecules, metabolism of xenobiotics by cytochrome P450, drug metabolism, and tight junction.

### 4.4. Correlation Matrices

The correlation matrix for each biological pathway was computed using the NumPy Python package. These matrices were then visualized using the pandas and matplotlib Python packages. Positive and negative correlations were represented in blue and red, respectively. Correlation plots for the top 10 biological pathways were then plotted for cancer patients and normal controls (Figure 1). Gene pairs showing a change in correlation of |ΔCC| ≥ 0.70 (significance cutoff level: 0.7) were identified and analyzed.

## 5. Conclusions

In this study, we integrated differential gene expression and gene expression correlation to identify a panel of 71 genes that may work together in concert and could potentially be used for the early diagnosis of CRC. Further studies including in vitro and in vivo validations are needed to confirm the roles they play both individually and coherently in the development of CRC. The major limitations of the current study are the small sample size and the lack of sufficient pathological information for the patients, which may decrease the sensitivity and specificity of our research protocol. We are currently endeavoring to establish collaborations with oncologists in both Canada and foreign countries in order to improve and optimize this potential CRC diagnostic panel of genes. Furthermore, we will conduct more detailed analysis of CRC in the future by including patients’ clinical information, such as tumor stage, tumor grade, and metastatic status.

## Figures and Tables

**Table 1:** Top 20 upregulated and downregulated DEGs in the colon cancer dataset TCGA-COAD.

Upregulated			Downregulated		
Gene Name	Log_2_FC ≥ 2.50	*p*-Value	Gene Name	Log_2_FC ≤ 2.50	*p*-Value
*MAGEA3*	11.81	0	*APOA4*	−8.12	0
*MAGEA6*	10.71	0	*OTOP2*	−7.95	0
*IGFL1*	10.60	0	*APOC3*	−7.84	0
*PRSS56*	10.55	0	*SLC10A2*	−7.16	0
*MAGEA12*	10.16	0	*APOA1*	−7.10	0
*KLK6*	9.74	0	*MS4A10*	−7.07	0
*PAEP*	9.67	0	*AQP8*	−6.86	0
*NOTUM*	9.65	0	*CA1*	−6.58	0
*PRR9*	9.51	0	*APOB*	−6.41	0
*KLK8*	9.28	0	*INSL5*	−6.14	0
*SPRR2E*	9.21	0	*TMIGD1*	−6.07	0
*PPBP*	9.21	0	*GUCA2B*	−6.06	0
*SPRR1A*	9.10	0	*G6PC*	−6.02	0
*MAGEA1*	9.07	1.58 × 10^−10^	*CPO*	−5.83	0
*FEZF1*	8.99	0	*KRTAP13-2*	−5.75	0
*DKK4*	8.93	0	*PYY*	−5.74	0
*ZIC5*	8.91	3.71 × 10^−13^	*OTOP3*	−5.73	0
*KLK7*	8.87	0	*BEST4*	−5.70	0
*CST1*	8.58	0	*SLC30A10*	−5.57	0
*SPRR2D*	8.52	0	*CLDN8*	−5.55	0

**Table 2:** Top 10 significant biological pathways identified for the upregulated and downregulated DEGs (|log_2_FC| ≥ 2.50) using InnateDB.

	Pathway Name	Uploaded Gene Count	Total Number of Genes
1	Neuroactive ligand–receptor interaction	29	275
2	Systemic lupus erythematosus	28	126
3	Cytokine–cytokine receptor interaction	27	258
4	Pathways in cancer	19	329
5	Bile secretion	18	72
6	Wnt signaling pathway	17	140
7	Cell adhesion molecules	17	144
8	Metabolism of xenobiotics by cytochrome P450	16	73
9	Drug metabolism	14	67
19	Tight junction	14	135

## Data Availability

Not applicable.

## Supplementary Materials

The following supporting information can be downloaded at: https://www.mdpi.com/article/10.3390/ijms232012463/s1.
